# Assessment of Soil Contamination with Potentially Toxic Elements and Soil Ecotoxicity of Botanical Garden in Brno, Czech Republic: Are Urban Botanical Gardens More Polluted Than Urban Parks?

**DOI:** 10.3390/ijerph18147622

**Published:** 2021-07-17

**Authors:** Vaclav Pecina, Martin Brtnicky, Marie Balkova, Jitka Hegrova, Martina Buckova, Tivadar Baltazar, Roman Licbinsky, Maja Radziemska

**Affiliations:** 1 Faculty of Chemistry, Institute of Chemistry and Technology of Environmental Protection, Brno University of Technology, Purkynova 118, 612 00 Brno, Czech Republic; xcpecina@fch.vut.cz; 2 Department of Agrochemistry, Soil Science, Microbiology and Plant Nutrition, Faculty of AgriSciences, Mendel University in Brno, Zemedelska 1, 613 00 Brno, Czech Republic; tivadar.baltazar@mendelu.cz; 3 Department of Geology and Soil Science, Faculty of Forestry and Wood Technology, Mendel University in Brno, Zemedelska 3, 613 00 Brno, Czech Republic; marie.balkova@mendelu.cz; 4 Transport Research Centre, Lisenska 33a, 636 00 Brno, Czech Republic; jitka.hegrova@cdv.cz (J.H.); martina.buckova@cdv.cz (M.B.); roman.licbinsky@cdv.cz (R.L.); 5 Institute of Environmental Engineering, Warsaw University of Life Sciences, 159 Nowoursynowska, 02-776 Warsaw, Poland

**Keywords:** urban green spaces, ornamental garden, soil toxicity, construction and demolition waste, contamination, risk assessment

## Abstract

Though botanical gardens are an important and widely visited component of urban green spaces (UGS) worldwide, their pollution is rarely studied. The aim of this study was to assess botanical garden soil contamination and ecotoxicity and to evaluate whether urban botanical gardens are more contaminated than urban parks. Soil assessments showed serious contamination with Cd, Pb and Zn, emitted predominantly by traffic, agrochemicals and past construction and demolition waste. The discovery of hazardous historical ecological burden in the UGS calls for the necessity of detailed surveys of such areas. Despite prevailing moderate-to-heavy contamination, the soil was only slightly ecotoxic. Maximum immobilisation inhibition of *Daphnia magna* reached 15%. Growth of *Sinapis alba* L. was predominantly stimulated (73%), and *Desmodesmus subspicatus Chodat* was exclusively stimulated, possibly due to soil alkalinity and fertiliser-related nutrients. The hypothesis of a higher contamination of urban botanical gardens compared to urban parks was confirmed. However, urban parks can face a greater risk of soil ecotoxicity, hypothetically due to decreased activity of soil organisms resulting from adverse soil conditions caused by active recreation. The results highlight the need for an increased focus on botanical and ornamental gardens when assessing and managing UGS as areas potentially more burdened with contamination.

## 1. Introduction

Urban soil pollution by anthropogenic activities is an important research topic [1,2]. Reduced soil quality poses a risk to human health and urban ecosystems [3,4,5,6]. Typically, soil in industrial areas or near roads contains the highest pollution levels due to the continuous emission of potentially toxic elements (PTEs) [7,8]. Industry, traffic [9,10] and coal combustion [4,10] emit high concentrations of PTEs and represent the most important sources of pollution in cities.

Urban green spaces (UGS) and their soils are an essential component of urban ecosystems [3,11,12]. They improve city dwellers’ living conditions through functions such as climate regulation, sustaining biogeochemical cycles, water flow regulation, runoff mitigation and pollutant retention [6,11,13,14,15]. UGS are often considered less polluted [16,17] and sometimes even used to determine background values for environmental contamination assessment [18,19]. However, due to their potential to retain and store pollutants, they may become hotspots of accumulated pollution. PTEs bind to the soil organic matter [1] of UGS and remain persistent in soil [20].

When assessing UGS pollution with PTEs, great attention is paid to public urban parks (e.g., Setälä et al. [6]; Urrutia-Goyes et al. [21]; Gu et al. [22]; Brtnický et al. [23]; Han et al. [24]). However, research rarely focuses on arboretums, botanical and ornamental gardens [10,25], despite their worldwide prevalence in urban areas and importance to local communities.

Botanical gardens and arboretums represent artificial but stable environments [26]. They were first introduced in the 16th century. There are 1775 registered in 148 countries according to the current definition of Botanic Gardens Conservation International [27], and more than 3000 have been registered according to the previous definition [28]. In addition to their focus on scientific research, conservation and education, botanical gardens and arboretums are popular tourist sites visited by approximately 500 million visitors per year [27] with a strong social relevance [29,30]. Cavender and Donnelly [13] even urge their increased involvement with urban forestry to improve sustainability of cities and human lives.

The greater focus on urban parks research may be due to residents’ higher frequency of visits, thus increasing the risk of exposure to soil pollutants. Additionally, the possibility of direct contact with contaminated soil is usually less likely in botanical gardens owing to the different nature of activities. Active and regular management of botanical gardens through fertilisers and various pesticides [9,25,26], on the other hand, can result in a higher risk of soil contamination. For example, Orecchio [31] found botanical garden soil contaminated with polycyclic aromatic hydrocarbons. Similarly, the potential of a higher load of PTEs in ornamental gardens was indicated by Biasioli et al. [32]. Martín et al. [10] even refer to botanical gardens as excellent tools to evaluate pollution. Thus, despite the lower risks to visitors, botanical gardens can hypothetically be more hazardous contamination hotspots of PTEs in urban areas.

To verify this hypothesis, several sub-steps were performed: (1) assessment of soil contamination with PTEs (As, Cd, Cr, Cu, Ni, Pb and Zn) in the botanical garden, (2) assessment of the spatial distribution of PTEs, (3) assessment of the soil ecotoxicity and (4) comparison of the contamination of botanical and ornamental gardens to urban parks.

## 2. Materials and Methods

### 2.1. Study Site

The studied botanical garden (and arboretum) is located in Brno, Czech Republic, at an altitude of 220–250 m a. s. l. in an area with an average total precipitation of approximately 550 mm/year and average annual temperature of 8.4 °C. The soil is primarily clayey with a high CaO content or artificial batch formed after clay mining. The arboretum was founded in 1938 on two hectares and was extended to eleven hectares in 1967. Until then, the area had a predominantly agricultural use. The lower section of the garden was adjacent to the construction landfill, and demolition waste remained after the bombing of the city during World War II. The northern section was bordered by an allotment and an agricultural area. Currently, a university campus, a dormitory, a sports area, an unmanaged green space, roads and tram tracks surround the garden. The garden grounds include an administrative building with classrooms, greenhouses and water features. It serves as a purpose-built educational facility and is also used by students for recreation and relaxation.

### 2.2. Soil Sampling and Laboratory Analysis

**Figure 1:**
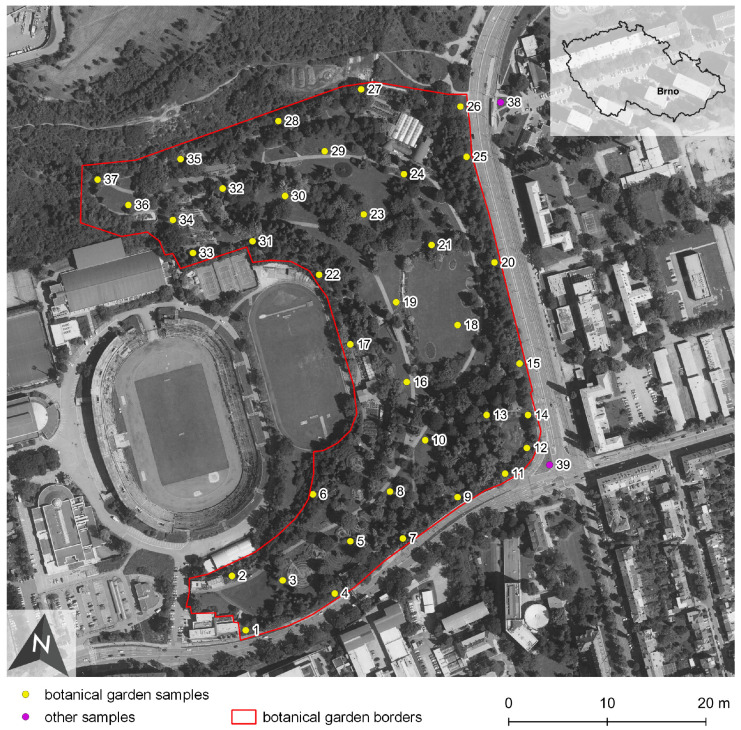
Study site with sampling points in the context of the surrounding urban area.

A total of 37 samples were collected from the botanical garden topsoil (0–5 cm), and two comparative samples were collected near the road and tram stops outside the garden. The samples were taken from the regular sampling network with a 5 m spacing (Figure 1). Three subsamples within a radius of 0.5 m from the sampling point were collected and subsequently mixed into one composite sample. Each composite sample consisted of 500–1000 g of soil. The samples were dried at room temperature and sieved through a nylon sieve (2 mm mesh size) for water extraction and then milled to a fine fraction in an oscillatory mill for the aqua regia extraction.

For trace element analysis, about 1 g of dry milled soil sample was digested in *aqua regia* mixture (prepared from subboiled ultrapure acids) in a high-pressure, high-temperature microwave digestion system SW-4 (Berghof, Eningen, Germany). The procedure followed ISO 11466:1995 [33]. The digested samples were diluted to a final volume of 100 mL with ultrapure water (Merck Millipore, Darmstadt, Germany). The digestion blank was prepared in parallel and was subtracted during evaluation.

Elemental determination was performed using a triple quadrupole inductively coupled plasma mass spectrometer ICP-MS/MS (Agilent Technologies, WaldBronn, Germany). Selected isotopes ^75^As, ^52^Cr, ^60^Ni, ^65^Cu, ^66^Zn, ^111^Cd and ^208^Pb, were measured in O_2_ reaction gas (0.29 mL/min) and He collision gas (4 mL/min) modes. The forwarded RF power was 1550 W, with a carrier gas flow rate of 1.07 L/min and integration time per isotope was 0.3 s. A calibration range of 0–100 µg/L for all elements was prepared by mixing from a 1.000 g/L single standard stock solution (Analytika, Prague, Czech Republic) in a matrix of 2% HNO_3_ (ultrapure) and 0.35% HCl (ultrapure). The internal standard solution was prepared in a final concentration of 100 µg/L as a mixture of Bi, Ge, In, Li, Sc, Tb, Y. Quality control was performed using reference materials (I) SRM 1640a Trace Elements in Natural Water (National Institute of Standards & Technology, Gaithersburg, MD, USA) for instrument settings and calibrations and during measurement for control of signal stability, and (II) QCM Metranal 33 Clay-loamy soil (Analytika, Prague, Czech Republic) with a certified content of elements leachable by the *aqua regia* used for control of the whole procedure (digestion and analysis). 

For ecotoxicological characterisation, water extraction in a Reax 20 rotary shaker (Heidolph Instruments, Schwabach, Germany) was carried out using 10.0 g of dry sieved soil sample and 100 mL of ultrapure water (Merck Millipore, Darmstadt, Germany) for 24 h at laboratory temperature. Extracts were subsequently centrifuged by Universal 320 R benchtop centrifuge (Hettich, Kirchlengern, Germany) for 20 min at the rotation speed of 4000 rpm and then filtered through a 5 µm paper filter. The filtered aqueous extracts’ pH was measured on a laboratory pH/Conductometer Orion 4 Star with a glass combination electrode. The pH measurement was performed according to ISO 10523:2010 [34].

Total organic carbon (TOC) was measured using a TOC analyser soli TOC^®^ cube (Elementar Analysensysteme GmbH, Langenselbold, Germany).

### 2.3. Soil Contamination Assessment

The soil contamination assessment was carried out by Geoaccumulation Index (I_geo_), which assesses soil contamination by comparing the current and expected pre-industrial PTE contents. The index is calculated as follows [35]:

(1) $$I_{\mathrm{geo}}=\log_{2}\left(\frac{C_{i}}{1.5B_{i}}\right)$$

where Ci represents the content of the PTE, 1.5 represents the constant reflecting natural fluctuations of the PTE content and Bi represents the content of the corresponding PTE in the background. The background values of Cd, Cu, Pb and Zn were taken from the study by Brtnický et al. [23], who conducted research in a nearby park (Luzanky Park). To determine the background values for As, Cr and Ni, two soil samples from a depth of 100 cm were taken and analysed from the same locality. The I_geo_ classes are as follows [2,26]: I_geo_ ≤ 0: uncontaminated; 0 < I_geo_ ≤ 1: uncontaminated to moderately contaminated; 1 < I_geo_ ≤ 2: moderately contaminated; 2 < I_geo_ ≤ 3: moderately to heavily contaminated; 3 < I_geo_ ≤ 4: heavily contaminated; 4 < I_geo_ ≤ 5: heavily to extremely contaminated; I_geo_ > 5: extremely contaminated.

### 2.4. Soil Ecotoxicity Assessment

Soil toxicity was assessed using acute ecotoxicity tests on three selected organisms representing different trophic levels: water planktonic crustacean *Daphnia magna*, freshwater green alga *Desmodesmus subspicatus* Chodat and the seeds of *Sinapis alba* L. The tests were performed on aqueous extracts of soil samples to evaluate the effect of water-leachable substances from soils on the organisms because of the greater importance of the bioavailable fraction of PTEs compared to the total soil PTE contents for risk assessment [36].

The *D. magna* crustacean test carried out according to ISO 6341:2013 [37] determined the effect of aqueous soil extracts on mortality and immobilisation of the organism. The test was run for 48 h at 20 °C, without aeration, light or feeding. The age of the tested organisms was a maximum of 24 h at the time of testing.

The test on the alga *D. subspicatus* was performed in microtiter serological plates according to ISO 8692:2012 [38]. Aqueous extracts of soils inoculated with algae and control populations (nutrient solution with added algae) were incubated by shaking them in a thermostat under constant illumination of 8000 lux at 23 °C for 72 h. Cell density conversion was used to calculate algal growth rates. Algal growth inhibition was calculated by comparing algal growth rates in aqueous soil extracts to the control population’s growth rate.

Tests on *S. alba* seeds were performed according to the Methodical Instruction of the Ministry of the Environment of the Czech Republic [39]. The test consisted of culturing the seeds under standard conditions in Petri dishes on filter paper saturated with aqueous soil extract and assessing the effect on seed germination and root growth. The plates were cultured for 72 h in a thermostat without light at a temperature of 20 °C. Inhibition or stimulation of root growth for a given sample was calculated by comparing the average root length in plates containing the test sample of aqueous soil extract against the average root length in control plates.

### 2.5. Spatial and Statistical Analysis

A vector point layer was created with the attribute table containing information on As, Cd, Cr, Cu, Ni, Pb and Zn soil contents. These sampling points were arranged into a regular net with a grid size of 0.1 × 0.1 km. Interpolation data analyses were performed using QGIS Desktop 3.4.13 software. Spline [40,41] was chosen as the geostatistical method for spatial interpolation. More specifically, the method with b-spline refinement and 0.0001 threshold error was selected. The spatial resolution of calculated output rasters was 0.1 m. These rasters were interpreted into colourful images based on quantile distribution into ten value classes.

Additional data processing and advanced statistical analysis were carried out using the statistical program R version 4.0.2. [42]. Multiple linear regression analysis was performed for modelling the relationship between the species immobilisation or inhibition with the dependence of I_geo_ of PTEs. The strength of the linear relationship between the PTEs and pH and TOC was measured by Pearson’s correlation coefficient. The principal component analysis (PCA) was implemented for reducing the number of variables of PTEs in used datasets. The comparison between the botanical garden and the park was made using the Tukey Honest Significant Difference (Tukey HSD) test at *p* = 0.05.

## 3. Result and Discussion

### 3.1. Soil Contamination Assessment

The results of the soil contamination assessment in the botanical garden are presented in Table 1. All the median values were below the limits [43], indicating an overall low level of pollution and risk. However, due to the very low background values, serious contamination is evident from the assessment using I_geo_ in some PTEs. Although As content levels were categorised as ‘uncontaminated’, indicating low enrichment from coal combustion [44,45], Ni, Cr and Cu levels ranged from ‘uncontaminated’ to a ‘moderately contaminated’ category. Cd content measured as ‘moderately contaminated’, Pb was ‘moderately contaminated’ to ‘heavily contaminated’ and Zn measured as ‘heavily contaminated’ to an ‘extremely contaminated’ category.

**Figure 2:**
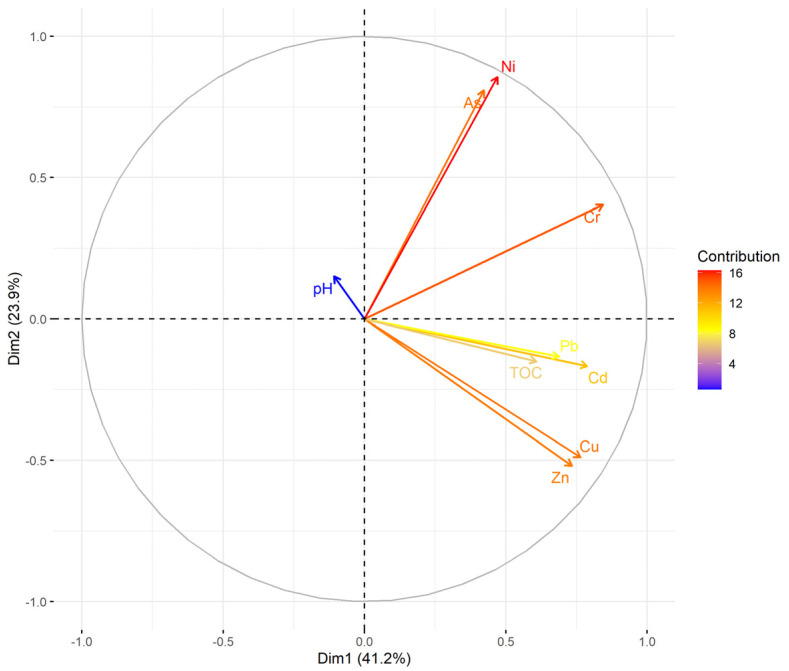
PCA biplot of pH, TOC and PTEs.

A similar grouping of the elements as in the I_geo_ assessment was also presented in the PCA (Figure 2). As, Ni and Cr formed one group of strongly correlated elements (Table 2) with low I_geo_ values, indicating a predominantly geogenic origin. The second group consisting of Cd, Pb, Cu and Zn was affected by the anthropogenic activities due to the increased I_geo_ values (Table 1). The overall low importance of soil pH (Figure 2) can be attributed mainly to the soil alkalinity (pH 7.8–9.0, average 8.2); PTEs relationships with pH were insignificant (Table 2). Positive correlations of PTEs with TOC (TOC 2.2–18.1%, average 5.7%) (Table 2) demonstrated their binding to soil organic matter [1].

**Figure 3:**
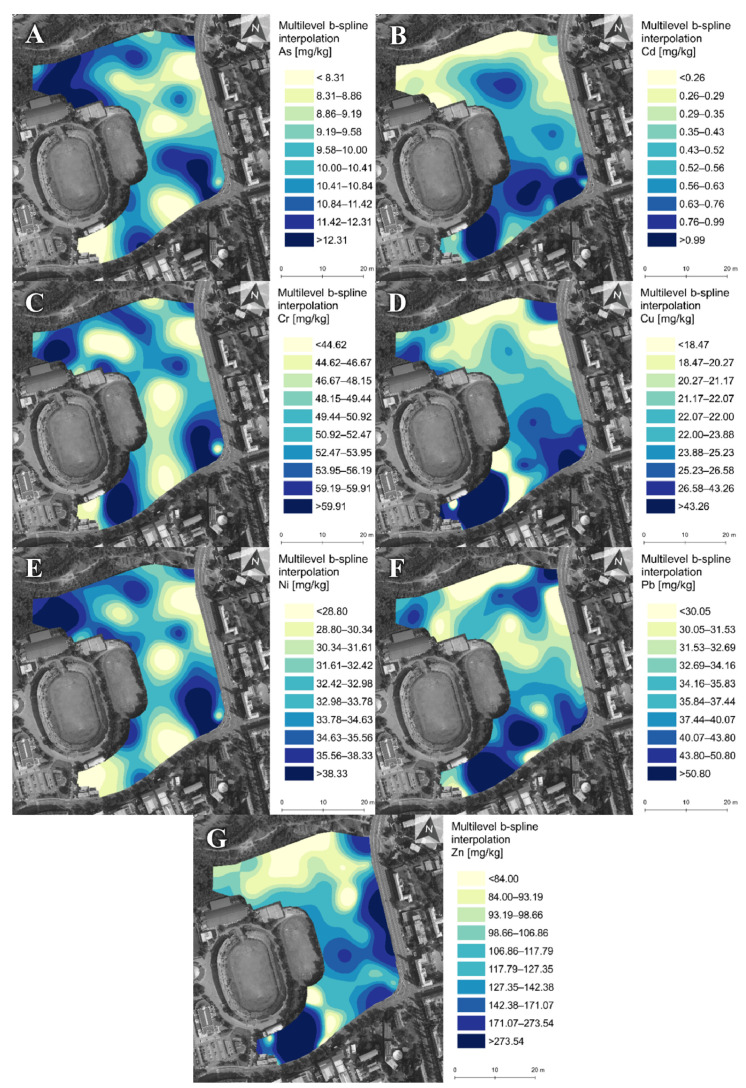
Spatial distribution of As (**A**), Cd (**B**), Cr (**C**), Cu (**D**), Ni (**E**), Pb (**F**) and Zn (**G**) in the botanical garden soils.

The results of the spatial distribution (Figure 3) showed mutual spatial relationships. All the PTEs reached increased values in the southeast part of the botanical garden near a busy crossroad. Such places are highly contaminated with PTEs and other pollutants due to stop-and-go traffic [17,19,46,47]. Road and tram traffic are a likely source of this enrichment with PTEs as one of the main emitters of urban contamination [5,19,48,49]. Road traffic is a primary source of Cd, Cr, Cu, Ni, Pb and Zn [46,47], and tram or train traffic emits a similar spectrum of metals [50]. Malkoc et al. [49] reported that tram traffic-related contamination does not reach levels as high as road levels, but the combination causes the highest contamination. The contamination originating from traffic is also indicated by slightly increased PTE values (especially Pb) in the referenced traffic sites compared to the garden corresponding median values. A significant correlation of Pb with Zn (Table 2) may be associated primarily with leaded petrol’s historical use [10,48]. The strong relationship between Zn and Cu is attributed to their presence in deteriorating brake components [5].

There is a considerable increase in Cd, Cr, Cu, Pb and Zn values in the southwest part of the botanical garden. The extreme values above DSG Intervention Value (Table 1, Cu and Zn reached maximum levels) indicate serious pollution. The origin of this pollution is probably underlying construction and demolition waste. A similar situation of botanical garden contamination by waste material is mentioned by Bretzel and Calderisi [48]. Cachada et al. [5] referred to increased Cu, Pb and Zn contents in ornamental gardens, potentially originating from previous industrial activity. These findings highlight the importance of UGS investigation and the history of their surroundings, as UGS can be seriously polluted by historical and often forgotten burdens.

Other elevated PTE levels (Figure 3) corresponded with more intensively managed zones of the botanical garden (especially in the northwest), signalling possible origin from agricultural chemicals. In addition to Cu and Zn, originating from the traffic, a strong correlation indicates their common source from fertilisers containing these elements as essential nutrients supporting plant growth. Historically, due to limited or non-existent legislation, applications of fertilisers and various pesticides were uncontrolled and could have contributed to soil contamination. Such contamination was reported by Apostoae [25].

Comparison to other botanical gardens is limited in scope due to the low number of studies presenting PTE values (Table 1). However, available evidence shows similar values without extreme deviations, indicating overall low pollution levels according to DSG Intervention Values [43].

### 3.2. Soil Ecotoxicity Assessment

Due to the low PTE values below the soil quality standards (Table 1), no ecotoxicity risk could be expected. However, soil is a complex system, and its ecotoxicity reflects the toxicity of all present substances, not only PTEs. Soil ecotoxicity is an expression of both synergic and antagonistic contaminants’ effect on organisms, their interactions upon the soil matrix and upon tested organisms [51,52]. Thus, chemical analysis results do not necessarily correlate with the results of ecotoxicological tests, specifically in soil where the degree of pollution is not very high [51].

**Figure 4:**
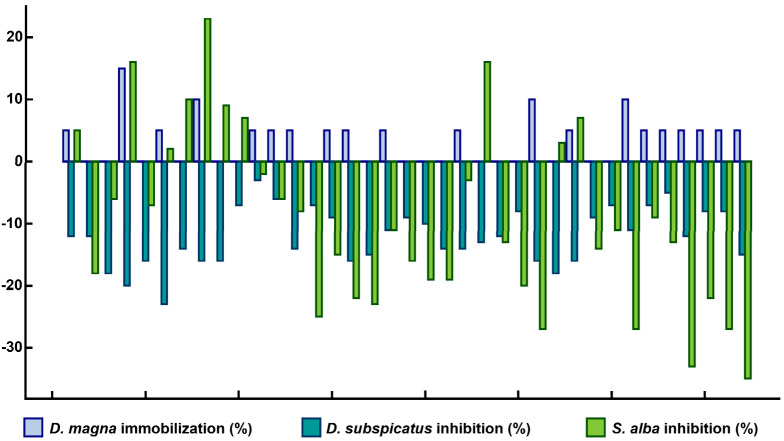
Soil ecotoxicity assessment using *Daphnia magna*, *Desmodesmus subspicatus* and *Sinapis alba*; negative values indicate growth stimulation.

Maximum immobilisation inhibition of *D. magna* reached 15% and was only 0–5% in 89% of cases (Figure 4). Increasing contamination with Ni (I_geo_ > 0), despite its overall low level, led to the immobilisation of *D. magna* in more than 70% of cases as it is a highly toxic element to crustaceans [53,54]. Continued contamination with Ni can be expected to negatively affect the vitality of crustacean populations in the botanical garden water features. There were only growth stimulation effects recorded in the test using algae *D. subspicatus* (Figure 4). Therefore, the contamination of the botanical garden does not pose a serious risk to aquatic organisms.

Similarly, slight toxic effects were recorded in the phytotoxicity test using *S. alba* (Figure 4). The plant growth was stimulated in 73% of cases and maximum growth inhibition reached 23%. Thus, the soil contamination posed only a slight phytotoxic risk for plants growing in the botanical garden.

Soil ecotoxicity assessment indicated a low level of PTEs-related threat. However, this threat may be slightly underestimated due to the alkaline nature of the soils. Metal bioavailability is a key factor in determining the metal’s toxicity and uptake by organisms [54] and is significantly affected by soil properties such as pH [48]. Such alkaline soil as in this case limits the presence of fractions of water-soluble soil contaminants in aqueous soil extracts [55]. Therefore, the overall PTEs bioavailability could be reduced, and the results showed decreased toxicity effect on the tested organisms. The frequently observed stimulation of both algae and *S. alba’s* growth may be due to the higher content of nutrients in the soil originating from fertilisers. Aruoja et al. [56] state that the harmful effect of PTEs on organisms can be masked in soils containing a high concentration of nutrients/supplements.

### 3.3. Comparison of Contamination of Urban Botanical Gardens and Urban Parks

**Figure 5:**
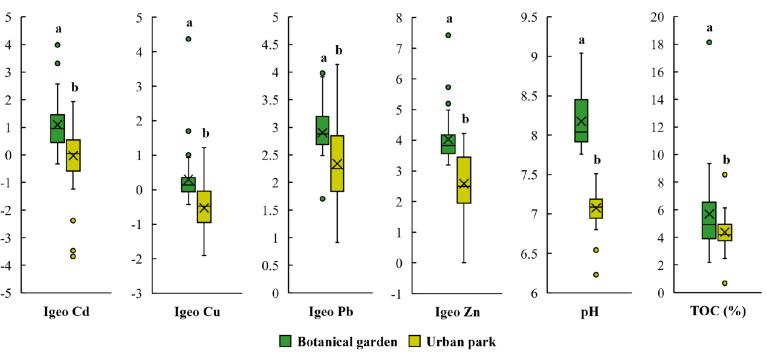
Comparison of PTE contamination (using Geoaccumulation Index, I_geo_), pH and TOC of botanical garden and urban park topsoil; different letters indicate significant differences at *p* = 0.05, Tukey HSD test.

To verify the hypothesis of higher soil contamination in botanical gardens compared to urban parks, the results were compared with the neighbouring park studied by Brtnický et al. [23]. Despite the negligible difference in distance, the botanical garden soils were significantly more contaminated with Cd, Cu, Pb and Zn (Figure 5). This result is consistent with other available studies (Table 3). In the botanical garden in Iași, Cd and Cu contents were 4.7× and 2× higher than in the nearby urban park.

Higher PTE values were found in the botanical garden even though the park was approximately 150 years older with the potential for more prolonged accumulation of PTEs and higher soil contamination levels [6,7,16]. The park is also surrounded by busier roads which were previously identified as primary sources of contamination. Therefore, the dominant reason for the higher contamination of the botanical garden is likely its management. Maintenance practices, including the use of various fertilisers and sprays, are also mentioned by Imperato et al. [9], Ruiz-Cortes et al. [57], Biasioli et al. [32] and Rodrigues et al. [1] as a source of contamination in square and ornamental gardens. Furthermore, the application of organic amendments and related significantly higher organic carbon content in the botanical garden topsoil compared to the park (Figure 5) could induce higher retention of PTEs [1] and their accumulation. The results of this and other studies (Table 3) confirm the hypothesis of higher contamination of botanical gardens compared to urban parks and attribute more intensive management as the primary cause.

Although the botanical garden was significantly more contaminated with PTEs (Figure 5), its soil was significantly (*p* = 0.05, Tukey HSD test) less phytotoxic than in the park. The garden soil’s phytotoxicity was found in only 27% of samples with a maximum inhibition value of 23% (Figure 4). In the park, 80% of samples with phytotoxicity reached 93% [23]. The lower toxicity of the botanical garden soil despite higher soil contamination may be attributed to higher activity of soil organisms. Organisms living in the soil of botanical gardens are not exposed to the extreme conditions (e.g., soil degradation due to trampling) experienced in public urban parks intensively used for recreation. More stable and favourable conditions can stimulate soil organisms’ activity and reduce the toxicity or even degrade some pollutants [58]. This correlates with the results presented by Lorenz and Kandeler [59], who found higher microbial biomass and microbial activity in urban gardens with natural mixed substrates on loess compared to urban public parks. However, further large-scale research is needed on this topic to verify this hypothesis. Another reason for the lower soil toxicity in the botanical garden may be the higher nutrient content resulting from using fertilisers which could diminish the possible toxic effects. This hypothesis is consistent with the stimulation of green algae and *S. alba* growth (Figure 4). Finally, significantly higher pH in botanical garden soil compared to the park pH levels (Figure 5) could limit the concentration of PTEs in aqueous extracts and reduce their bioavailability for the tested organisms.

Trees are critical components of UGS and their protection ensuring that people gain the benefits trees provide should be priority [13]. The absence of such stresses for vegetation induced by soil toxicity may contribute to a more effective provision of ecosystem services [20] of botanical gardens compared to urban parks. As a result, the importance of botanical gardens and arboretums may be higher and more beneficial to public health. On the other hand, elevated PTE levels in the soil can be riskier for regular botanical garden visitors. The comparison and evaluation of benefits and potential threats should be the subject of further research.

## 4. Conclusions

Though the botanical garden soil assessments indicated serious contamination with Cd, Pb and Zn, all PTE contents were below the soil quality standards. The sources of contaminants were predominantly from traffic, historically deposited construction and demolition waste and agrochemicals. The soil does not pose a severe ecotoxicological risk to aquatic organisms, although the content of Ni, as a potentially hazardous element for crustaceans, should be monitored. Prevailing growth stimulation of the *S. alba* indicated only slight soil phytotoxicity. Though few studies exist with similar findings and results are often disregarded, the hypothesis of a higher contamination of botanical and ornamental gardens compared to urban parks was confirmed. Because its indications appear globally, more attention needs to be paid to urban botanical and ornamental gardens as UGS potentially more burdened with contamination. Soil ecotoxicity of the botanical garden was low and not as risky as in the neighbouring urban park. The reasons for this need a more detailed investigation. 

## Figures and Tables

**Table 1:** PTE contents (mg/kg) in botanical garden soils, garden surroundings, standards (DSG = Dutch Soil Guidelines), contamination assessment (I_geo_) and PTE contents in other gardens (mean values in mg/kg).

Data	As	Cd	Cr	Cu	Ni	Pb	Zn
Average S.D. Minimum	10.3	0.61	52.2	37.7	33.8	38.1	172
	1.99	0.61	8.32	69.8	4.91	13.4	219
	6.68	0.18	38.1	16.3	25.0	15.6	75.6
Median	10.2	0.43	50.3	24.0	33.4	35.4	117
Maximum	14.7	3.51	75.3	453	47.2	75.8	1414
Reference Traffic Sites Background	13.7	0.40	65.3	29.6	39.6	52.5	132
	6.92	0.15 ^1^	29.3	14.6 ^1^	21.3	3.2 ^1^	5.5 ^1^
DSG Target Value ^2^ DSG Intervention Value ^2^	29	0.8	100	36	35	85	140
	55	12	380	190	210	530	720
I_geo_	−0.04	1.09	0.23	0.30	0.05	2.90	4.03
Iași Botanical Garden ^3^	12.4	0.36	55.5	73.5	54.8	33.8	113
Harbin Botanical Garden ^4^	-	0.14	73.5	34.3	-	30.3	119

^1^ Brtnický et al. [23]; ^2^ VROM [43]; ^3^ Apostoae [25]; ^4^ Meng et al. [8].

**Table 2:** Correlation matrix of the soil PTE contents; * significant at *p* = 0.05; ** significant at *p* = 0.01; *** significant at *p* = 0.001.

As	0.09	0.64 ***	0.01	0.83 ***	0.22	−0.03	0.01	0.01
	Cd	0.54 ***	0.53 ***	0.25	0.45 **	0.48 **	−0.09	0.74 ***
		Cr	0.50 **	0.75 ***	0.43 **	0.46 **	−0.08	0.34 *
			Cu	−0.06	0.53 ***	0.95 ***	−0.13	0.30
				Ni	0.18	−0.11	0.11	0.19
					Pb	0.50 **	−0.16	0.29
						Zn	−0.01	0.34 *
							pH	0.01
								TOC

**Table 3:** Comparison of urban parks and botanical (or ornamental) gardens PTE contents (mg/kg). Lowercase letters indicate significant differences between their PTE contents at 0.05 (Tukey HSD test) when comparing urban gardens and urban parks in the PTE contents with non-available values: H = Higher content and L = Lower content.

City	Botanical Garden				Urban Park				Justification
	Cd	Cu	Pb	Zn	Cd	Cu	Pb	Zn	
Brno	0.61 a	37.7 a	38.1 a	172 a	0.28 ^1^b	16.6 ^1^b	27.2 ^1^b	59.0 ^1^b	See the text
Iași	0.36 ^2^	73.5 ^2^	33.8 ^2^	113 ^2^	0.08 ^3^	37.5 ^3^	46.7 ^3^	217 ^3^	N.A.
Naples ^4^	-	H	H	H	-	L	L	L	Park soils were better protected from contamination.
Ljubljana, Sevilla and Torino ^5^	-	H	H	H	-	L	L	L	Park soils were less affected by anthropogenic disturbance.
Beijing ^6,^*	H	H	H	H	L	L	L	L	Historical use of PTEs in gardens.
Sevilla ^7^	-	H	H	H	-	L	L	L	Use of organic amendments in gardens.

^1^ Brtnický et al. [23], ^2^ Apostoae [25], ^3^ Apostoae et al. [28], ^4^ Imperato et al. [9], ^5^ Biasioli et al. [32], ^6^ Xia et al. [4], ^7^ Ruiz-Cortes et al. [57], * Significant (*p* < 0.05).

## Data Availability

The data presented in this study are available on request from the corresponding author.
